# Socially facilitative robots for older adults to alleviate social isolation: A participatory design workshop approach in the US and Japan

**DOI:** 10.3389/fpsyg.2022.904019

**Published:** 2022-10-19

**Authors:** Marlena R. Fraune, Takanori Komatsu, Harrison R. Preusse, Danielle K. Langlois, Rachel H. Y. Au, Katrina Ling, Shogo Suda, Kiko Nakamura, Katherine M. Tsui

**Affiliations:** ^1^Department of Psychology, New Mexico State University, Las Cruces, NM, United States; ^2^Department of Frontier Media Science, Meiji University, Tokyo, Japan; ^3^Toyota Research Institute, Cambridge, MA, United States

**Keywords:** social robots, human-robot interaction, experience-grounded participatory design, older adults, US, Japan, social isolation, cross-cultural study

## Abstract

Social technology can improve the quality of older adults' social lives and mitigate negative mental and physical health outcomes associated with loneliness, but it should be designed collaboratively with this population. In this paper, we used participatory design (PD) methods to investigate how robots might be used as social facilitators for middle-aged and older adults (age 50+) in both the US and Japan. We conducted PD workshops in the US and Japan because both countries are concerned about the social isolation of these older adults due to their rapidly aging populations. We developed a novel approach to participatory design of future technologies that spends 2/3 of the PD session asking participants about their own life experiences as a foundation. This grounds the conversation in reality, creates rapport among the participants, and engages them in creative critical thinking. Then, we build upon this foundation, pose an abstract topic, and ask participants to brainstorm on the topic based on their previous discussion. In both countries, participants were eager to actively discuss design ideas for socially facilitative robots and imagine how they might improve their social lives. US participants suggested design ideas for telepresence robots, social distancing robots, and social skills artificial intelligence programs, while Japanese participants suggested ideas for pet robots, robots for sharing experiences, and easy-to-operate instructor robots. Comparing these two countries, we found that US participants saw robots as tools to help facilitate their social connections, while Japanese participants envisioned robots to function as surrogate companions for their parents and distract them from loneliness when they were unavailable. With this paper, we contribute to the literature in two main ways, presenting: (1) A novel approach to participatory design of future technologies that grounds participants in their everyday experience, and (2) Results of the study indicating how middle-aged and older adults from the US and Japan wanted technologies to improve their social lives. Although we conducted the workshops during the COVID-19 pandemic, many findings generalized to other situations related to social isolation, such as older adults living alone.

## 1. Introduction

Loneliness and social isolation lead to negative mental and physical health, particularly for independent-living older adults (Perissinotto et al., 2012; Cudjoe et al., 2020). The quality of intimate relationships is a strong predictor of the wellbeing of older adults (Lehr et al., 2009). Without quality close relationships, older adults feel lonelier, resulting in poorer physical and mental health (e.g., Fees et al., 1999). Importantly, older adults care more about the quality rather than the quantity of their social networks (Pinquart and Sorensen, 2001). However, older adults may face social isolation for various reasons, including domestic isolation, limited social contact, and social disengagement (Philip et al., 2020).

Although many existing online communication and robotic technologies can improve the social lives of older adults (Wada et al., 2003; Banks et al., 2008; Chen et al., 2018; Pu et al., 2019), some older adults hesitate to adopt new technologies (Barnard et al., 2013). This is true even when they are unable to meet others in person because of their health, difficulty traveling, being far from family, or other reasons. Social technology intended to improve the quality of older adults' social lives and mitigate negative mental and physical health outcomes associated with loneliness should be designed collaboratively with this population to both ensure that they will use it and that it will actually improve the quality of their social lives (Gustafson et al., 2016).

In this paper, we present (1) a novel participatory design (PD) workshop in which we grounded participants in their actual experience and (2) results in which we (A) uncovered older adult's challenges with their current social lives and technology use and (B) developed concepts for socially-facilitative robots to address their needs. Specifically, we conducted the PD workshops in US and Japan because both countries are concerned about the social isolation of these older adults due to the rapid aging of their populations (Magnus, 2012; Goldin, 2016; Götmark et al., 2018), and share a strong commitment to robotics and some fundamental psychological regularities; but they differ in a number of other respects, like culture, traditions and social norms.

Previous work on social robots for older adults has often studied the benefits of robots offering companionship and communication (Pinquart and Sorensen, 2001; Chen et al., 2018 for reviews). Little research examined how robots enhance human-human social interaction. Our study adds to this literature. Notably, it took place during a widely-enforced period of social isolation due to the COVID-19 pandemic. These unique circumstances increased the relevance of social isolation's negative effects on mental health, and the importance of developing solutions to mitigate this issue. However, social isolation was recognized as a “serious public health concern” (Cacioppo and Cacioppo, 2014) prior to the pandemic, and will likely remain so even after the social isolation caused by the pandemic is no longer in effect.

## 2. Related work

### 2.1. Social isolation and health

Social isolation, defined as low quantity and quality of social and emotional connection (Shankar et al., 2011), is associated with increased feelings of loneliness along with numerous negative physical and mental health outcomes (Weiss, 1973; Van Baarsen et al., 2001; Wang et al., 2003; Tomaka et al., 2006; Shankar et al., 2011). Loneliness is the negative affective state experienced when an individual feels a discrepancy between the social relationships they wish to have and those they perceive they have (Heinrich and Gullone, 2006). Loneliness correlates with negative mental health, including increased social anxiety, higher risk of depression, suicidal ideation, decreased cognitive function, and poor life satisfaction (Heinrich and Gullone, 2006; Hawkley and Cacioppo, 2010). Loneliness also predicts negative physical health, like poor cardiovascular health (e.g., high blood pressure, high cholesterol), and risky health behaviors (e.g., smoking, physical inactivity) (Hawkley and Cacioppo, 2010; Shankar et al., 2011).

Social isolation and loneliness are of particular concern among older adult populations. National data of the United States showed that 24% of adults over 65 years of age considered to be socially isolated, and 43% of adults over 60 report feeling lonely (Perissinotto et al., 2012; Cudjoe et al., 2020). The mental and physical effects of social isolation and loneliness among older adults are especially pronounced. These factors correlate with more frequent doctor visits, more rapid onset of cognitive decline and Alzheimer's disease, and risk of all-cause mortality among the older adults (Wilson et al., 2007; Gerst-Emerson and Jayawardhana, 2015; Boss et al., 2016; Beller and Wagner, 2018).

### 2.2. Social isolation and COVID-19

While the effects of social isolation and loneliness on older adult populations has been a public health concern for some time, societal responses to the COVID-19 pandemic brought these issues to the forefront (Gerst-Emerson and Jayawardhana, 2015; Armitage and Nellums, 2020). COVID-19, formally known as SARS-CoV-2, is a virus which originated in Wuhan, China in December 2019, before spreading globally. By July 2021, nearly 200 million people globally had been infected by COVID-19 (Hopkins, 2020). By March 2022, over 450 million people had been infected (Worldometer, 2022).

In response to the rapid spread of the virus, governments around the world ordered their citizens to observe various degrees of physical distancing (i.e., keeping physical distance between an individual and people who do not live in the same household), from restrictions on international travel to mandatory stay-at-home orders (Giallonardo et al., 2020; González-Rodŕıguez and Labad, 2020; Moreland et al., 2020). Restrictions on socializing in person increased the prevalence of mental health conditions like anxiety and depression in global populations during the COVID-19 pandemic (Lytridis et al., 2020; Saladino et al., 2020; Sikali, 2020). Older adults had higher mortality from COVID-19, leading to recommendations that family members avoid contact with the older adults to “keep them safe.” Further, traditional social outlets such as places of worship were closed, which may have put older adult populations at disproportionate risk of negative mental health outcomes (Armitage and Nellums, 2020; Tyrrell and Williams, 2020).

### 2.3. Social technology and socially facilitative robots

Current and emerging technologies may help older adults maintain social connections while physically separate from others. Use of social technology, like e-mail, online video calls, and social media, correlated with better self-reported health, fewer chronic illnesses, and reduced depressive symptoms among older adults (Chopik, 2016; Lee et al., 2021), because these social technologies helped reduce loneliness (Chopik, 2016), especially during social isolation (Fraune et al., 2022). However, people view technology-mediated communication as more psychologically demanding and enjoy it less than in-person interaction (Williams, 2021).

Novel robotic technology may provide a solution to challenges with technology in communication. Robots can have unique socially facilitative effects on interactions between multiple users in many settings. In human-robot cooperation tasks, human-robot teams with a robot member revealing its vulnerabilities were more likely to laugh together, console teammates who made a mistake, and communicate well than teams with a robot members not revealing its vulnerabilities (Strohkorb Sebo et al., 2018). Similarly, a robot that made vulnerable statements about itself also led their human team members to converse more, distribute speaking time more equitably, and rate their group more positively overall (Traeger et al., 2020).

Socially facilitative robots are also widely used for companionship and communication to improve the social life of older adults. In eldercare facilities, the therapeutic seal robot PARO became a common topic of conversation, connecting both older residents and their caregivers (Wada et al., 2003). Interacting with PARO also increased the density of older adults' social networks (Wada and Shibata, 2007) and reduced depressive symptoms in older adults (Chen et al., 2018).

The robotic dog, AIBO, acted a social companion of residents in long-term aged care facilities and reduced loneliness of older adults (Banks et al., 2008). While online communication and social robots may help connect older adults with friends and loved ones, some older adults may hesitate to rely upon novel technologies. Many older adults expressed difficulty using many modern communicative technologies (Ling et al., 2022). Despite the benefits of technologies on the quality of life of seniors aged 65 and older (Heinz et al., 2013), about half of this age group did not currently use the internet and believed that it did not put them at a disadvantage. Seventy-seven percent of seniors reported that they required assistance when learning to use new technology (Smith, 2014). Reflective of this preference for older technology, many nursing home residents preferred letters and phone calls over video chats during the COVID-19 pandemic (Fearn et al., 2021). Social technology intended for older adults should be designed collaboratively with this population, both to ensure that they are willing and able to use it, and that the technology can improve the quality of their social lives and subsequent mental and physical health.

### 2.4. Cultural differences between US and Japan

Cross-cultural studies in Human-Robot Interaction (HRI) have documented varying attitudes toward robots across the US, Japan, the Netherlands, China, Mexico, and Germany (Bartneck et al., 2005). They have explored what assumptions people across Japan, Korea, and the US make about humanoid and animal-type robots (Nomura et al., 2008) or about humanoid and product-like robots (Lee et al., 2015). Other studies have explored people's acceptance of robots across cultures (Banks et al., 2008; Cacioppo and Cacioppo, 2014; Beller and Wagner, 2018), and on cultural impact on the credibility of robot speech in US and Arabic communities (Andrist et al., 2015). However, up to now, no studies have examined to conduct the participatory design workshops for exploring robot design concepts in different two countries.

In this study, we focused on two cultures: the US and Japan. These two countries are concerned about the social isolation of these older adults due to the rapid aging of their populations (Magnus, 2012; Goldin, 2016; Götmark et al., 2018), and share a strong commitment to robotics and some fundamental psychological regularities; however, they differ in a number of other respects, most notably in their broad social-cultural values [i.e., collectivism vs. individualism (Harry Hui and Triandis, 1986; Triandis et al., 1988)], religious traditions (Buddhism and Shintoism in Japan, Judeo-Christian traditions in US), and public views of robots (Nitto et al., 2017). These differences may affect people's different perceptions and different expected roles of the robots in their daily environment.

### 2.5. Participatory design

Participatory design (PD) methods involve users as active collaborators, alongside traditional researchers and designers, throughout the process of designing new technology and improving existing technology to ensure that their needs are met (Lee et al., 2017). Through PD, users first self-identify their own needs and wants, then help co-design technological solutions to meet those needs. A PD approach benefits both researchers and users through mutual learning. End users have the most direct understanding of how they approach technology and the environments in which they use it (Lee et al., 2017). Using PD techniques, designers can learn from users' experiences to develop technology that takes into account these social use contexts. Meanwhile, users learn about the current capabilities and applications of state-of-the-art technology, and they gain insight into the design process itself.

The PD approach has gained popularity among HRI researchers as a way to understand the perspectives of diverse users and stake holders in a variety of contexts. The Neighborhood Networks project was interested in how an urban community in Pittsburgh, PA, would think to use robotic and environmental sensing technologies in their own neighborhood (DiSalvo et al., 2008). In summer 2007, researchers engaged the local community in a series of PD activities over the course of several weeks including a scavenger hunt activity using sensing technologies, storyboarding their concepts, “open studio” prototyping, and “science fair” presentations; although 20 residents initially participated, ultimately, 12 participants presented to each other and another 25 members of their neighborhood, including a city planner. In the summer and fall of 2014, Šabanović et al. (2015) conducted a series of 2 PD workshops with five older adults with co-occurring major depression and chronic physical illness (3m, 2f; ages 58–71) in conjunction with interviews of 5 staff members of a large outpatient healthcare provider; the goal of the project was to understand how Socially Assistive Robots (SARs) could be designed for and used in the homes of older adults before they become institutionalized. In 2020, Georgiou et al. (2020) conducted a PD workshop with 10 stroke survivors (7m, 3f; *M* = 58 years, *SD* = 12.4) to explore how SARs could assist stroke survivors with self-managed rehabilitation. As noted by Mason (2010), qualitative studies, including PD workshops, require a longer time and therefore tend to have lower sample sizes; correspondingly, the outcomes are qualitative in nature. Although replication is important in general (Amrhein et al., 2019), statistical testing is not always appropriate in the case of the small number of subjects (Gliner et al., 2002) and it is better to listen to a small number of actual users that the researchers can obtain and interview than to have no representation of these users at all.

Previous work has shown that PD workshops were particularly valuable and empowering for older adults, which is especially important for this group because they are traditionally marginalized in technology development (Lee et al., 2017; Laura Raḿırez Galleguillos and Coşkun, 2020). Older adults are often stereotyped as less willing to use new technology, but these effects are accounted for by their experience with technology (Ezer et al., 2009; Flandorfer, 2012). Because older adults have difficulty envisioning intangible concepts and future technologies, it is imperative to customize the PD method that engages older adults' creative thinking (Lindsay et al., 2012). The work in this paper examines how robots can facilitate middle-aged and older adults' social interactions with friend and family outside of the home. Robots still have not yet become ubiquitous in domestic settings, so it is unlikely that people, particularly older adults, have no or minimal experience with robots or robotics technologies. Direct discussing robots at the start of a PD session would force people to draw on second-hand sources, such as science-fiction books, movies, and documentaries, or purely speculating. To preempt this, in our PD approach, we dedicated 2 of the 3 rounds of discussion to people's own life experiences, thereby both grounding the conversation in reality, creating rapport among the group, and engaging them in creative critical thinking about robots in a grounded manner. With that foundation, then we engage the group to discuss how a robot or robotic technology might be able to help address their previously discussed challenges.

This work of course is not the first occurrence of using PD approaches to investigate how robots can help older adults; for example, Šabanović et al. (2015) researched how socially-assistive robots might fit into older adults' daily home lives by providing them directly with social interaction and companionship. Our study adds to these findings by examining how robots can facilitate middle-aged and older adults' social interaction with friends and family outside the home.

## 3. Materials and methods

This method was developed for PD groups in the US and then modified with respect to culture for the PD groups in Japan^1^. We conducted PD to (A) identify middle-aged and older adults' challenges with current technology-mediated social interactions, and then (B) brainstorm socially-facilitative robot concepts to address their stated needs and wants.

### 3.1. Design teams

We recruited in total seven participants to join the PD workshops in US, and in 12 participants in Japan; due to the global pandemic, the PD sessions were virtual (Feil-Seifer et al., 2020). In each workshop, there were two facilitators and two to three participants. Facilitators were researchers on the current study with a background in technology research and design, while participants had no background in research and design. As part of the prior interview, participants viewed a video of current commercial robots to ensure all participants in the PD had a common understanding of the capability of current robotic technology, as opposed to drawing from movies (Sundar et al., 2016). During each session, one facilitator led the discussion and another mainly took notes. The facilitator leading the workshop posed all questions and brainstorming prompts to the participants and moderated the discussion. The note-taker paraphrased participant comments and themes of the discussion on a shared screen throughout the workshop.

#### 3.1.1. Participant inclusion criteria and recruitment

Our work started during the global pandemic, which limited our contact with older adults in our community due to the obvious health risks. The term “older adult” is typically used to describe people age 65 years or older, which is the ideal target population of our research. Some previous work using participatory design in the HRI literature has also used older adults aged below 60 (e.g., Šabanović et al., 2015) and similarly, there is evidence of wider age bands regarding research on smartphones (e.g., Gao et al., 2015), Internet use (e.g., Morrell et al., 2000; Sum et al., 2008), and care technologies for activities of daily living (e.g., Itoh et al., 2021). We therefore have adjusted our recruitment requirement to older adults aged 50 or above.

The inclusion criteria for these PD workshops was that a person was: (1) 50+ years of age, (2) able to use video conferencing software; also participants were either (3a) a resident of the US and spoke English, or (3b) a resident of Japan and spoke Japanese. The average age of the US participants was 59.29 years (*SD* = 5.85) and 55.42 years in Japan (*SD* = 4.17); demographic information of all participants is displayed in Table 1. Our sample includes current older adults (age 65+) and also people who are contemplating their upcoming retirement (middle-aged people 50+).

**Table 1:** Demographic information of the participants (P#_*country*_).

**US**				**Japan**			
**Participant**	**Age**	**Gender**	**Workshop session**	**Participant**	**Age**	**Gender**	**Workshop session**
P1_*US*_	58	Man	3	P1_*Japan*_	50	Woman	1
P2_*US*_	57	Woman	1	P2_*Japan*_	56	Woman	1
P3_*US*_	51	Woman	2	P3_*Japan*_	55	Man	1
P4_*US*_	62	Man	1	P4_*Japan*_	53	Man	2
P5_*US*_	68	Woman	1	P5_*Japan*_	50	Woman	2
P6_*US*_	64	Man	2	P6_*Japan*_	58	Woman	2
P7_*US*_	54	Woman	3	P7_*Japan*_	57	Woman	3
				P8_*Japan*_	63	Man	3
				P9_*Japan*_	50	Man	3
				P10_*Japan*_	57	Woman	4
				P11_*Japan*_	54	Man	4
				P12_*Japan*_	62	Man	4

To recruit participants in the US, we posted an invitation to the study to Reddit.com and the researchers' personal social media page; the invitation contained basic descriptions of the study and eligibility to participate. We also recruited participants from prior related studies (Fraune et al., 2022; Ling et al., 2022) who had indicate that they wanted to be recontacted for future studies. We then used snowball sampling to increase to sample size. Participants in Japan were recruited through a human resource dispatching company. The compensation was $70 USD and ¥7,000 JPY per participant in the US and Japan, respectively.

### 3.2. Procedure

The PD workshops in the US took place on November 2, November 10, and December 4, 2020, and the workshops in Japan took place on August 29 and September 5, 2021. Due to restrictions on in-person interactions during the COVID-19 pandemic, all workshop sessions in both countries occurred via Zoom online video chat. All PD sessions were video recorded. The researchers mailed participants study supplies (post-it notes, markers) prior to the workshop. Each session lasted approximately 75 min.

Each workshop session consisted of three rounds, and each focused on a different main theme. All rounds began with a 5-min brainstorming phase, then used a ‘round-robin' discussion format, with the facilitator allowing each participant the opportunity to share an initial idea one at a time before opening the floor to a more freeform discussion. This ensured that each participant had the chance to contribute their ideas during the early stages of each round, so that the following discussion was informed by the opinions of all present. Through all rounds, the note-taker paraphrased participant comments and main ideas on Google Slides, using it as a shared ‘digital whiteboard' to provide a common reference point for continued discussion.

*Round 1* consisted of an initial discussion of the types of technologies participants currently use to communicate with others. The facilitator encouraged participants to consider both what they liked about social experiences facilitated by technology, and what aspects of their technology-mediated interaction were missing or altered when compared to in-person interactions.

*Round 2* centered on a review of the social challenges participants currently faced, such as keeping up with old friends, making new connections, or socializing at large gatherings. Because the workshops occurred during the COVID-19 pandemic, the facilitator welcomed participants to share both new challenges specific to the unique social circumstances of the time and general challenges that existed before social distancing norms.

*Round 3* involved a robot design session. The facilitator instructed participants to brainstorm ideas for robots to help solve one of the social challenges discussed in Round 2. The facilitator further encouraged participants to focus on ideas for a robot they would personally want to own and use and would be technically feasible within the next 3 years. After the initial brainstorm and discussion, the facilitator instructed participants to pick one of the ideas discussed (either their own or another participant's) to improve upon, or add to, in a subsequent 3-min brainstorm session. This gave participants the opportunity to participate in iterative design. Another round-robin discussion followed this second brainstorming session. When appropriate, the facilitator re-focused the discussion or posed high-level questions, such as “What problem is the robot solving?” and “What might be some challenges of that idea?” Finally, the facilitator asked participants to create a list of their five favorite robot features discussed in this round.

**Figure 1:**
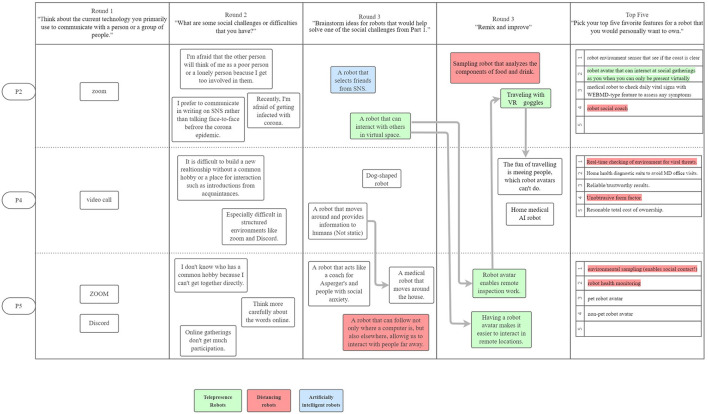
The first US PD session was held on November 2, 2020 and had 3 participants. The light arrows indicate concepts shared between participants. ”Telepresence robots” and ”distancing robots” were highly discussed.

**Figure 2:**
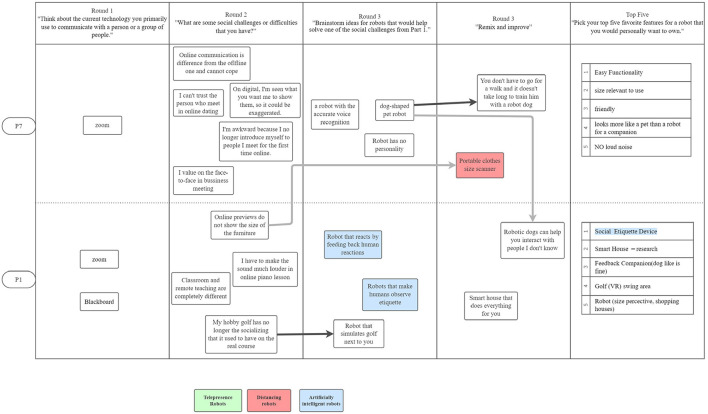
The second US PD session was held on November 10, 2020 and had 2 participants.

**Figure 3:**
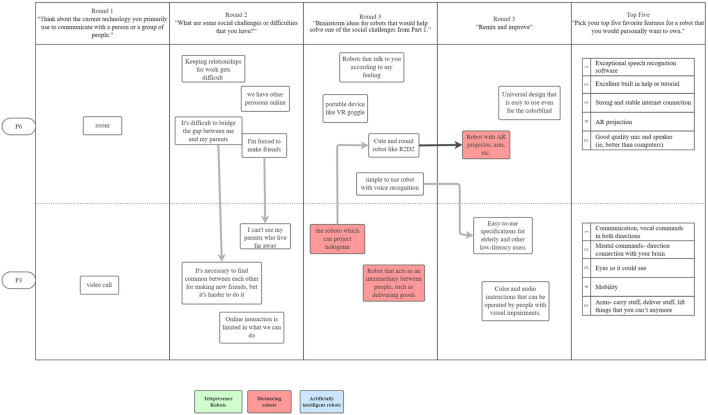
The third US PD session was held on December 4, 2020 and had 2 participants.

Figures 1–3 are visualizations of each US PD session, and **Figures 5**–**8** are visualizations of each Japanese PD session; these figures provide an overview of flow during the PD sessions. The red, green, and blue shading in the figures call out the top 3 concepts from each country, and arrows show how the concept evolved. The dark black arrows indicate concepts that participants created and then carried forward their idea for development. The light gray arrows indicate that another participant either further developed the concept or had a similar concept.

### 3.3. Analysis

We transcribed video-recordings of each workshop session. Researchers then analyzed and coded these transcripts along common themes. Themes were derived from open and axial coding (Glaser et al., 1968); the coding scheme is available in the Supplementary materials. The same coding rubric was utilized in both the US and Japanese PD transcripts. Interrater agreement ranged from moderate to strong in US (IRRs > 0.67) and strong in Japan (IRRs > 0.75) (Miles and Huberman, 1994).

## 4. Results

We identified some major themes to discuss. We break them into topics that parallel the discussion rounds: (1) Benefits and drawbacks of existing communicative technologies, (2) Challenges maintaining existing and developing new social relationships, and (3) Ideas for designing robots to enhance their social lives. Below we do so first for US and then Japan PD sessions.

### 4.1. US findings

#### 4.1.1. Current technology use

In the first round of each workshop, participants discussed their current use of social technologies. Although they viewed these technologies as convenient and helping them connect with others, they also pointed out many drawbacks and problems of current technologies. More importantly, many participants considered online communication technologies lacking important aspects of in-person communication.

*1) Benefits of current technology*: Participants across all three workshops discussed various benefits of technology-mediated interactions. P1_*US*_ declared that a “bright spot” of social technology was that “at least I get to see my family” during COVID-19. P2_*US*_ echoed this sentiment in a separate session, indicating her gratitude for the ability to stay safely connected via video calls with her 85-year-old mother in a nursing home. Several participants discussed that video chat was preferable to texting or social media posts because they allowed some degree of non-verbal communication.

Some participants discussed their perception that not all technology-mediated social interactions are necessarily equal. P1_*US*_ contrasted a “pleasant” video call with family and friends with only two participants with a particularly overwhelming family video-chat experience where:

There's nine [virtual] screens there and one of them is on a phone and … the girlfriend's holding the phone in the car while the guy's driving and … just the whole kind of big thing. (P1_*US*_)

A participant in another session compared the more structured environment of Zoom, in which a user may already know everyone on the call, with more organic interactions on Facebook which has the potential to spark new friendships:

Friends of friends I see comment on my post, or I comment on their posts, and so you get at least a little bit of a relationship with them, and that can spin off into more of a friendship. (P4_*US*_)

P3_*US*_ shared her appreciation for the Discord messaging app, as it allows her to organize all her interests and clubs in one place, while giving her the choice of participating in synchronous (voice) or asynchronous (text) conversations. P1_*US*_, a teacher, also highlighted the advances educational software platforms made during the pandemic to more closely recreate a classroom experience, by adding features like a ‘raise hand' button.

*2) Drawbacks of current technology*: While participants acknowledged these benefits, the consensus of all seven participants across all three workshops, was that technology-mediated social interactions are inferior to their in-person counterparts. Criticisms of technology-mediated social interaction generally aligned with two major themes: (1) technical issues hinder the quality of time spent socializing online, and (2) social technology highlights integral missing aspects of face-to-face social interactions.

*a) Technical issues*: Several participants discussed technical issues with current social technology, which frustrate users over repeated interactions and ultimately limit this technology's usefulness. A common shared concern was the difficulty of teaching older or less technically proficient family and friends to use new platforms, with P2_*US*_ sharing a particularly frustrating experience when organizing a video call with her extended family, including her mother in a nursing home.

During two sessions, participants shared stories of social interactions abruptly ending due to dropped internet connections. P6_*US*_ elaborated that he expects technology to work when he needs it to, without the need to restart or troubleshoot it every time. P4_*US*_ noted that while “fuzzy” audio quality on video calls may not detract from his understanding of what someone else is saying, it adds social distraction that is absent from face-to-face interactions.

*b) Missing aspects from face-to-face interactions*: While the above-discussed technical issues may be addressed through software updates and usability testing, participants criticized technology-mediated social interactions as lacking many components of in-person communication. P6_*US*_ summarized this as the sense that “I'm just talking to a screen, rather than… a person…. Even though I can see the person's reaction, it's still not the same reaction that you get when you're in person.”

Almost every participant shared examples of essential features of social interaction that they felt were missing from current online platforms, such as intonation, a feeling of spontaneity, and a sense of shared space. Difficulties determining when it was one's turn to speak on video calls were discussed in two separate PD sessions, with P7_*US*_ stating that she felt “like I'm interrupting every time.” Several participants noted the challenge of conveying emotional tone when communicating via text:

When you talk to somebody, you can read their body language. You can see their facial expression; you can hear their intonation… You know whether or not they're like kidding around or if they're really emphatic, and so it's hard when I'm reading text to … know [if they are] … serious about what they're typing. (P5_*US*_)

Several participants discussed how losing a shared sense of space affected their social and business interactions. P4_*US*_ and P5_*US*_ shared a passion for attending science-fiction conventions, but noted that the energy was missing from online alternatives, with P4_*US*_ declaring virtual conventions “totally worthless.” P7_*US*_, a realtor, discussed the social difficulties she faces when showing homes to prospective buyers via video calls. She shared that a crucial skill for realtors is knowing how to discuss a home's minor flaws, such as chipped paint, in the context of its overall attractive qualities during tours. On virtual tours, however, she felt the need to focus her phone's camera on every minor defect in a home, which poses a novel social and professional challenge that she had not yet solved. Another participant (P2_*US*_) stated that she could not imagine trying to open Christmas presents with her family over Zoom, as video calls still “feel unreal” to her.

P3_*US*_ summarized participants' overall sense of the essential elements of in-person interactions that are missing from current technology: “I think… it's probably just camaraderie that you can mimic but not really recreate when you're not in person.”

#### 4.1.2. Current social challenges

In the second round of each workshop, participants discussed current social challenges they faced. While participants had difficulty maintaining existing social relationships, they spoke at greater length on the hurdles they faced when attempting to connect with new people.

*1) Challenges maintaining existing social relationships*: Participants across two sessions discussed challenges maintaining existing social relationships. P7_*US*_ shared how her general tendency to show up late to appointments with friends leads to stress as she worries about how long people will want to keep making plans with her. P3_*US*_ related the challenge of keeping her parents satisfied with the quality and quantity of their get togethers, especially during the COVID-19 pandemic. While she found it important to maintain a small, self-contained social network of family and friends during the pandemic, her parents did not, and subsequently struggled to understand why she was hesitant to visit with them in-person.

A few participants also recognized technical obstacles that challenged their ability to keep in contact with people close to them:

[Many online social opportunities are] based on relationships that were established before all this started and so … it somehow seems important to maintain those contacts, but it's more difficult unless you're very deliberate about it. (P5_*US*_)

Although P5_*US*_'s mother had already passed away prior to the pandemic, P5_*US*_ did not believe she would have been able to keep in contact with her mother during the COVID-19 pandemic or other situations in which in-person visits were not allowed, given the difficulty of teaching her mother how to use new technology.

*2) Challenges connecting with new people*: Across all three sessions, participants discussed troubles they encountered, both personality- and technology-related, when attempting to connect with new people. Two participants discussed their ongoing experiences of social anxiety, which requires them to make a conscious and sometimes exhausting effort to go to a party or reach out to new people. P7_*US*_ noted the challenge of picking her word choice carefully (even outside COVID-19 and online interaction) so as not to offend new people; she shared an example: ‘[realtors] can't say ‘master bedroom' anymore. It's now “owner's retreat.”'

The predominance of technology-mediated social interactions during the COVID-19 pandemic posed unique challenges. Participants across all three sessions shared that their most common methods of meeting new people (particularly other older adults) were currently unavailable, with P5_*US*_ concluding that it was “pretty much impossible” to meet new people during the pandemic.

Several participants noted that they typically connect with new people over common interests. However, as groups moved social interactions online during the pandemic (e.g., pre-planned Zoom calls between established social groups), it became harder to make new friends. For example, P5_*US*_ enjoyed meeting new people through a board game club prior to the pandemic. While she was able to socialize with existing members of her club through online meetups, she emphasized that this was not an adequate substitute, as new members were not able to attend club meetings during this time. In another session, P1_*US*_ appreciated that he was able to continue to play golf with a small group of friends, but missed having spontaneous interactions with strangers on the course, such as striking up an impromptu challenge between parties on the driving range or getting to know new people at the clubhouse bar.

P6_*US*_ suggested that the utility of social technology depends on the type of activity a group shares, with online meetups well-suited to ‘mental' activities (e.g., watching a movie, playing an online game), but incapable of meeting the needs of those more interested in ‘physical' activities (e.g., hiking).

P3_*US*_, a recent doctoral graduate, faced novel social challenges searching for a job during the pandemic. While she felt confident maintaining a professional network in the past, she currently faced the new challenge of establishing an online presence on LinkedIn and virtually connecting with peers and recruiters.

Two sessions featured discussions concerning the difficulty participants had building trust in people they meet online, beyond the time of COVID-19. P3_*US*_ highlighted that a person's online persona might not reflect who they actually are, while P7_*US*_ shared that she generally relies upon an introduction from an existing friend when building trust in a new person. Lacking this personal introduction, P8 expressed difficulty imagining how a person could establish trust in a potential partner met through an online dating service.

Overall, all except one participant expressed a clear preference for in-person social interactions, a sentiment summarized by P4_*US*_'s judgment of meeting people online being “much more laborious and drawn out [than] if you could sit down at a party over a beer and just chat.” The only participant who preferred technology-mediated interactions in some cases, P2_*US*_, shared that socializing online helped ease her social anxiety:

It's much easier to sit down and type and talk to people on Facebook through the written word than I've ever found it to go to a party … and having to speak to new people… It's not something I look forward to doing, whereas on Facebook… I can read something, decide if I want to respond at all, take my time to compose what I write as a response, and I like that security of a little bit of time to compose. (P2_*US*_)

#### 4.1.3. Robot design concepts

While initial ideas for robots varied widely, as participants refined their designs through the round robin and iterative design phases, their final concepts aligned along three themes: (1) telepresence robots, (2) distancing robots, and (3) artificial intelligence robots; Figures 1–3 show the conversation flow.

Below we discuss these concepts and then the limitations that robotic technology would still have.

*1) Telepresence robots*: Telepresence robots were the most discussed type, with six participants across all three sessions sharing their personal concepts of how this robot might facilitate social interaction. Participants expected a useful telepresence robot to attend physical social events in place of its user. They decided that the user should control it remotely and the robot should send audiovisual information to the user, allowing a user to have some degree of presence in a remote physical location.

P2_*US*_ discussed how she might use such a robot to attend the birthday party of a family member living on the opposite side of the United States, while P1_*US*_, an avid golfer, imagined a robot that could replicate movements (e.g., swings, putts) he made at home on a physically remote course. P5_*US*_ was the most enthused by the potential of telepresence robots, offering several possible applications, including for attending sci-fi conventions, business trips, and virtual tourism. The idea of virtual tourism, in particular, interested P2_*US*_ and P4_*US*_ in his session, sparking a discussion on how robots might allow one to virtually see the pyramids in Egypt, or avoid post-travel COVID-19 quarantine requirements.

Participants further developed how they envisioned telepresence robots in context of other current technologies during the iterative design phase of the study. P2_*US*_ and P5_*US*_ discussed the possibility of coupling a robot with a virtual reality (VR) interface, so the robot could transmit a full three-dimensional visual scene of its location, rather than just a 2D webcam feed. In another session, participants considered how robot design might include augmented reality (AR) technology, with P3_*US*_ and P6_*US*_ drawing inspiration from hologram communication in Star Wars. P3_*US*_ imagined a device that would “project [images of] people into space,” with P6_*US*_ agreeing that the added third-dimension to the conversation would “make it seem more real.”

**Figure 4:**
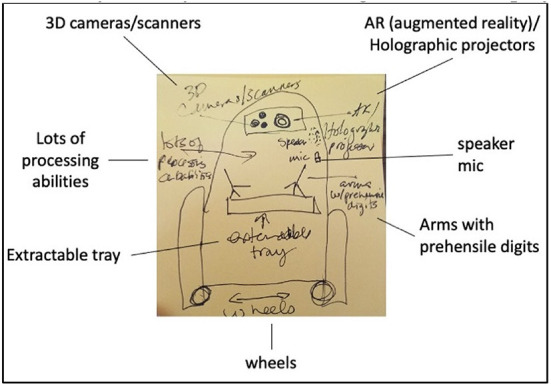
P3_*US*_ drew a robot concept and indicated its features.

*2) Distancing robots*: Four participants across two sessions developed ideas for a robot to facilitate social distancing during the time of the COVID-19 pandemic and other situations of limited physical contact. This robot would allow users to remain socially distant while engaged in activities that typically require physical proximity. P6_*US*_ imagined a robot that would transport shared items, such as a shared meal or a board game, between distanced users. P3_*US*_, the only participant who opted to sketch her robot design, imagined a robot that incorporated features from both the telepresence and social-distancing applications discussed during their session (Figure 4). Inspired by R2-D2, she imagined a “smaller” robot on wheels with a “great big processor” inside its body. Her robot would be equipped with a lens to project an augmented-reality conversation partner, 3D cameras, and speaker/microphone array to allow communication with remote users. In proximity with others, the robot used its arms with “prehensile grubby fingers” and an extendable tray to carry a shared board game between players.

*3) Artificially intelligent robot*: In two sessions, participants also considered concepts using artificial intelligence (AI) to improve the quality of their social interactions, but that would not necessarily require physical embodiment inside a robot. P2_*US*_ built on her understanding of Facebook algorithms to develop an idea for a “friend sorter” agent, which would connect strangers based on shared interests. In a separate session, P1_*US*_ considered an etiquette program for communicating on social media to ensure the tone and content of a person's posts was conveyed as intended. He imagined this as functionally similar to the spell-checking function in Microsoft Word, where the program may highlight a potentially problematic phrase for the user to reconsider. P7_*US*_ agreed that this concept may help her when she wants to avoid causing offense in her word choice.

*4) Limitations of robots*: While all participants contributed ideas for social robots, a common point of discussion across two sessions was participants' belief that, due to the inherent limitations of technology-mediated interactions, robots would not ultimately mitigate the challenges of their social interactions. Three participants' (P2_*US*_, P4_*US*_, and P5_*US*_) discussion on the potential of using telepresence robots for virtual tourism turned toward a discussion of the elements of traveling that they most valued, such as getting to know a new culture, and experiencing local scents and tactile sensations:

In certain applications and in the current (COVID-19) environment, that's helpful. But if I think about trips I've taken overseas or even within the US to interesting places, the strongest memories I have … are the people that I met… there. (P2_*US*_)

These participants concluded that the experience of travel consists of many intangible elements, beyond the visual sights, which technology could not adequately convey.

In another case, P2_*US*_ immediately qualified her idea of a “friend sorter” algorithm, with concerns:

Part of the divides in our country are from people becoming too insular and living in echo chambers, [so] a robot that… introduce[s] us to people who only think like us might make that sort of division worse. (P2_*US*_)

Another participant (P7_*US*_) shared that she still considered robots and virtual assistants to belong in the domain of movies, because real-world technology does not match the promise of stories told about them. She shared her frustrations of using the text-to-speech programs on her phone, and her desire for assistants to feature more fully-developed personalities:

To me, robots don't have personality… Personality is huge… people try to make Alexa have a personality by asking stupid question[s],… and they get back funny answers, but they don't make sense. (P7_*US*_)

P4_*US*_ held a more fundamental disagreement with the concept of proposing robotics as a means of facilitating social interaction:

Almost every near-term robotic…application you can think of … is more a substitute for human social interaction… so it would act to reinforce driving people apart because it makes it more comfortable to be apart. (P4_*US*_)

P4_*US*_ further shared his belief that “the only thing I could think of that would enable social interaction from a robotics point of view would be to develop … a real-time environmental sampling [robot].” In his conception, this robot would move through a crowd in traditional social environments such as bars or convention centers to determine if an area contained traces of a virus, therefore building confidence in a return to in-person socializing, especially after COVID-19.

Alongside discussing the roles socially facilitative robots might fill, participants shared critical attributes about if they would be willing to use a robot. P7_*US*_ shared concerns over privacy when using assistants like Alexa, while P4_*US*_ stated that he would not be interested in humanoid robots.

P3_*US*_ and P6_*US*_ valued the ease-of-use of any new technology:

Whatever it is, make it easy to use. Make it intuitive, so that the user doesn't really have to do anything… know how it works or… push a lot of buttons…. It just works. (P6_*US*_)

Both participants considered ease-of-use not merely a benefit, but essential for accessibility to people even older than them. P3_*US*_ considered if her parents would use a complex robot, given that using an iPhone or Kindle is already a challenge for them. P6_*US*_ further shared his accessibility concerns, stating that:

Especially if you're talking robots to aid [the] elderly… they're not going to be as technologically advanced…or…as mentally capable of handling the technology. (P6_*US*_)

These two participants considered speech recognition to be the most accessible way for older adults to interact with technology. However, P3_*US*_ noted her own accessibility concerns with existing speech recognition software when relating how Siri was sometimes unable to understand her father's slurred speech after he suffered a stroke.

### 4.2. Japanese findings

### 4.2.1. Current technology use

Like the US PD workshops, the middle-aged and older adult participants discussed their current use of social technologies and the benefits and drawbacks of these technologies. While they feel the convenience of communicating with people who are apart through the current social technologies, they also raised pain points that the amount of information they can acquire through these technologies was limited compared to in-person communication. It can be said that they unwillingly use such technologies when they cannot meet people in-person manner during COVID-19, and they strongly prefer in-person communication to technologically mediated ones.

*1) Benefits of current technology:* P6_*Japan*_ said about current technology-mediated communication, “I can send a message to my colleagues anytime and anywhere at my convenience without worrying about whether they are too busy to reply to me.” Because of the asynchronicity of text-based communication (not like in-person conversation), one can contact the others without worrying about their current status. P5_*Japan*_, P7_*Japan*_, and P8_*Japan*_ also said that the benefit of such technology-mediated communication is that they can send messages at their conveniences.

P7_*Japan*_ also remarked that the other benefit of such communication is to save everyone time because it does not require traveling to see the person in-person manner. COVID-19 has eventually led to widespread the use of video conference system so this made people eliminate the need to travel. In terms of less need to travel, the other participants also said that saving time to traveling to see the others makes it easier to manage the schedules of the online meetings and appointments.

Seven out of 12 participants answered that LINE, a freeware app for instant communications on electronic devices such as smartphones^2^, was their current preferred social technology. As for the reason, P1_*Japan*_ said, “I use LINE a lot. Because on this app, there is a display to show whether the message receiver has read the sent message or not, and this makes me intuitively understand whether she/he has time now or not.” Therefore, the display function whether the receiver has read the sent message or not on text-based social technologies will have a significant role in such asynchronous communication.

Another comment on the benefit of LINE was the integrated function of the phone call and video chat. P3_*Japan*_ commented, “There are very few people who do not install LINE on their smartphone, and you can do almost everything about communicating with others. You can make a phone call if you need, and you can also have a video chat especially when you want to see the face of that person, so this can be useful to communicating not only with my family but also with friends.” Some participants preferred such video chats over text-based communications because of rich amounts of information about the person in line including non-verbal information.

*2) Drawbacks of current technology:* While participants felt that such current social technologies are useful tool to connect people who are apart, they also felt that there are some issues of these technologies. These issues can be categorized into following two categories, like in US workshops: technological issues and missing aspects from face-to-face interactions.

*a) Technical issues:* P6_*Japan*_ mentioned, “There is often a huge time lag during video chat conversation, while there is no such lag during in-person conversation.” P11_*Japan*_ also said, “I still have some troubles with internet connections, like bad or lost connections.” The qualities of video chat conversation depend on the internet environments of users. Further, the more and richer information that is sent and received, the longer and slower it takes to transmit such information, which disrupts smooth communication like in-person ones.

Because online communication through current social technologies was created for one-on-one interactions, the conversations on these technologies tend to be limited on a single topic even if many people were in the conversation. P10_*Japan*_ said, “With Zoom, nobody can have a conversation with a large number of people at once. And this Zoom is inconvenient for having a light chat a kind of cross-talk among the colleagues. So the conversation on Zoom is different from ones in real life.” Some participants also pointed out that it is almost impossible using current social technologies for users to discuss multiple topics in parallel, like at a real party.

*b) Missing aspects from face-to-face interaction:* P1_*Japan*_ argued, “One of the significant differences between face-to-face and technology-mediated conversation is the amount of information that comes out from the person in LINE [app]. When you meet someone via in-person situations, you can see and sense their facial expressions and tones of voices. I cannot feel such non-verbal or paralinguistic information with the current social technology I am using now, so I guess this is the biggest differences.” Eight out of the 12 participants reported similar issues. In addition, P9_*Japan*_ said that he could not understand where the person using LINE is actually looking at him on their screen during the video chat, so he sometimes doubted whether they were concentrated on talking with him or if they were distracted and doing other things at the same time.

### 4.2.2. Current social challenges

As well as US workshops, participants discussed current social challenges they faced in terms of the following two aspects: maintaining existing social relationship, and attempting to connect with new people.

*1) Challenges maintaining existing social relationships:* Participants discussed the difficulties and problems they felt in maintaining their current relationships. P11_*Japan*_ said, “In my case, I can no longer have drinking parties or reunion parties I used to have every year. The adverse effect of this is that I have lost the trivial information that facilitates relationships.” As the result of COVID-19 pandemic, participants had fewer opportunities to meet their colleagues and friends in-person manner, so they lost out on opportunities to learn about current situation of these peoples. P3_*Japan*_, who mentioned a similar opinion with P11_*Japan*_, remarked that he started to contact these people more frequently than before the pandemic.

The most common comments in Round 2 were about how to maintain the relationships with their elder families, especially their parents, who are far away from them. Eight of 12 participants said that they felt difficulty in communicating with their parents, worried about their health and social situations, so they kept in touch with their parents more frequently than before COVID-19. In addition, there were several comments that their parents were too old to master the current technologies, so they usually use phone to communicate (voice call only). In terms of the use of current technology other than the phone by older adults, P7_*Japan*_ said, “It is difficult for older adults to master current video chat systems, so they tend to use the phone.” P11_*Japan*_ made assumptions about the reason why the older adults hesitate to use current technologies saying, “To master the current technologies, older adults have to get used to them, but this is quite hard, and they are somewhat reluctant to do so. This eventually leads their negative or passive attitude about new technologies.”

*2) Challenges connecting with new people:* Participants discussed their social challenges to connect with new people in terms of the following two aspects: human-caused challenges and technology-related ones. Regarding human-caused challenges, P3_*Japan*_ mentioned that he could no longer casually meet people via social networking services (SNSs), “Before the pandemic, I used to casually meet people who have similar hobbies through Twitter or the other SNSs, but now I cannot do that at all, so my social connection with others is completely text-based one.” P5_*Japan*_, who was working as a short-period temporary worker, said that she no longer has the opportunity to meet new people because the number of jobs has drastically decreased due to COVID-19. While many participants said that they passively lost their opportunities to establish new relationships, P1_*Japan*_ said that she was not actively trying to establish new relationship to prevent infections of COVID-19, “Because of this pandemic, I do not think I should go into the other's private too much, so I am not actively trying to meet new people.”

Next, as a technology-related challenges, P7_*Japan*_ said, “Because I only can see the other person's face or upper body in Zoom, it is difficult to capture a full picture of this person or their mood through the screen.” In this regard, P4_*Japan*_ and P9_*Japan*_ also said that it is difficult to establish new connections in on-line technologies because of the lack of information about the other person.

### 4.2.3. Robot design concepts

**Figure 5:**
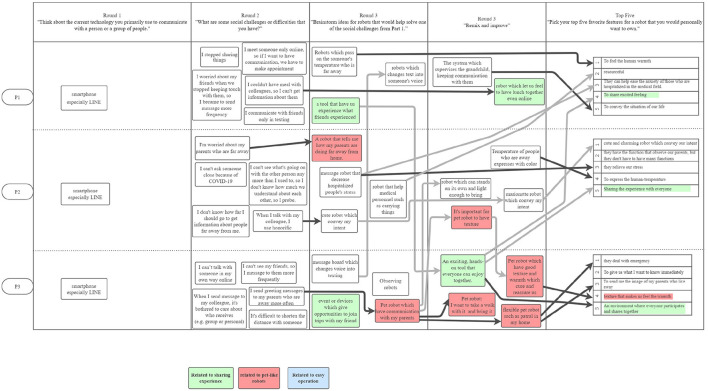
The first PD session in Japan was held on August 29, 2021 and had 3 participants. Dark black arrows indicate concepts that were carried forward in development. Two of the top themes were heavily discussed among the participants: sharing experiences and pet-like robots. All three participants chose a feature related to sharing experiences in their “top 5”.

**Figure 6:**
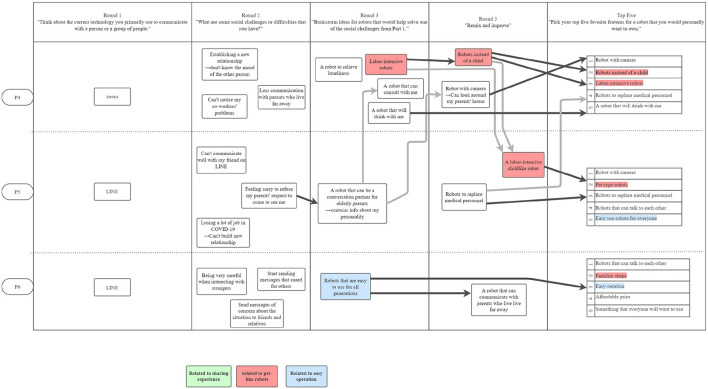
The second PD session in Japan was also held on August 29, 2021 and had 3 participants. Similarly, the theme of pet-like robots arose; “easy operation” was another top theme, as indicated by participants in their “top 5”.

**Figure 7:**
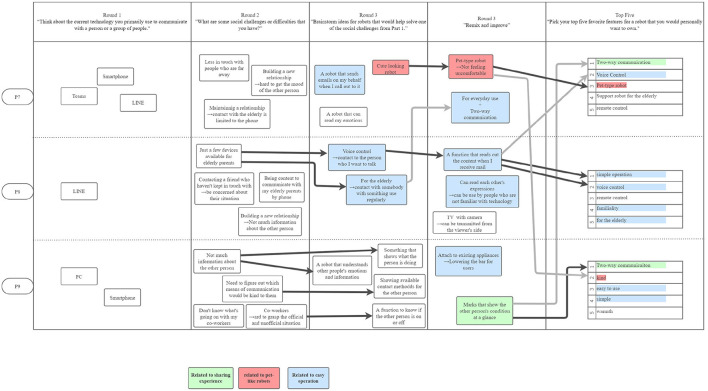
The third PD session in Japan was held on September 5, 2021 and had 3 participants. All participants discussed the need for easy operation. All three top themes were present in the participants' “top 5”: sharing experiences, pet-like robots, and easy operation.

**Figure 8:**
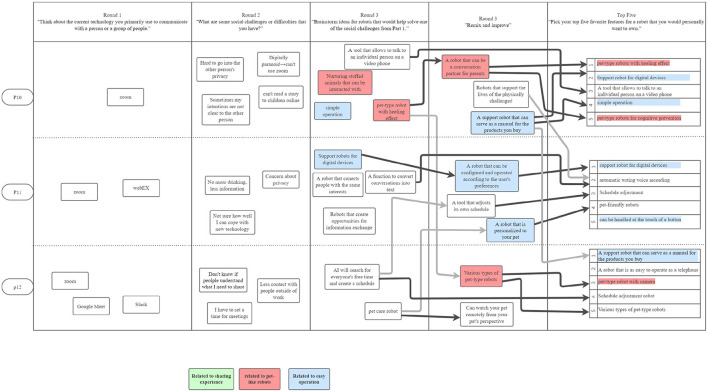
The fourth PD session in Japan was also held on September 5, 2021 and had 3 participants. P10 and P12 discussed pet-like robots, and P10 and P11 discussed easy operation. All three participants noted the importance of easy operation in their “top 5”.

The participants' final robot design concepts eventually settled on the following three themes: (1) pet robots, (2) sharing experiences, and (3) easy operation; Figures 5–8 show the conversation flow.

*1) Pet robots:* In Japanese PD workshops, the most common robot concept was a pet robot. In all four workshops, a pet robot was discussed, and nine out of 12 participants expressed their opinions about pet robots, as seen in Figures 5–8 (red). These participants mentioned in Round 2 that they were worried about their elderly parents who live apart, and many of them wanted to place a pet robot in their parents' house on the participants' to provide them with a social companion when they were unavailable to visit.

P2_*Japan*_ said, “The numbers of older adults is increasing, so robots that can watch over these older adults, including pet robots, can play a significant role in near future. These robots can be also applied to industries of nursing care; I hope the introduction of these robots will change the world eventually.” P10_*Japan*_ has already given the pet robot to her parents, and her mother happily reported that she is always talking to this robot on her (P10_*Japan*_) behalf. Participants expected pet robots to be useful in relieving the loneliness of parents who are apart and in communicating and dealing with problem when they occur.

As discussions on pet robots progressed, participants discussed not only the roles of the pet robots but also their required abilities or capabilities. Specifically, six participants mentioned the texture or appearance of the pet robots because all of these participants agreed that the pet robots commercially available today has a mechanical impression so they are looking for robots that are more like living things. P2_*Japan*_ said, “I think it would be great if the robot can feel the body temperature of a distant person, for example, this robot can tell the measured temperature by its color.” People are comforted by the presence and body heat of living things, like pets, so participants discussed that a more biological robot could be realized by incorporating such body warmth mechanism into a pet robot.

In addition, participants indicated that a significant element of the pet robots is to make the person invested in the robot by spending the time and effort on it. Although it is believed that robots are developed to reduce human's workloads by taking on their tasks, some participants said that the users would feel more attracted to their pet robots if they felt attached to and needed to care for these robots, like a *Tamagotchi* (Cheok and Zhang, 2019). P4_*Japan*_ said, “Pet animals are generally troublesome in that the owners should spend a lot of time and effort on them; on the other hand, such efforts make the owners feel more attached to them and more soothed on them.” The time and effort required to take care of a robot would be a significant factor in realizing a long-term interaction between the users and robots as well as between the owners and pet animals.

*2) Sharing experiences:* In two of four Japanese workshops, participants discussed how to improve sharing experiences with people who are apart. Due to the pandemic of COVID-19 mandates and social pressure to keep others safe, it became difficult to meet people in-person, and the conversations and drinking party using video conference or chat system have become popular. However, some participants felt that the quality of experiences is lower when communicating through such technologies compared to meeting in-person manner. P1_*Japan*_ said,

“I like to travel a lot, and I used to have a lot of travel friends before the pandemic. But now I cannot see them very often, so we use a kind of bulletin board system on the internet to share the information about travel. Currently, there are only text and some pictures on this bulletin board, so I hope there will be a tool that will allow people to experience what they have actually been through in real life.” (P1_*Japan*_)

As in the US workshops, participants discussed that there are a lot of issues especially in entertainment that are not feasible on-line manner, like the inability of traveling. In the Japanese workshops, although there were no concrete discussions about how the robots can resolve these issues, half of the participants said that they felt huge challenges of sharing experiences.

*3) Easy-to-operate instructor robot:* Participants reported some issues keeping up with rapidly evolving technologies. Many participants require some advice from the others who are good at current technologies when they face new devices or applications. However, they were concerned that they do not always have access to someone to assist them. P11_*Japan*_ said,

“When we have problems about current technologies, we usually ask friends or colleagues who are good at such technologies, but we do not always have such people around, and these difficulties derived from new devices or application are advancing day by day. It is difficult to keep up with all of these issues, so it would be very reassuring for the older adults (like us) if there were a robot with latest updated technological capabilities, and this robot beside us give instructions how to use these technologies.” (P11_*Japan*_)

He hoped that the robot will solve the situation in which there is no one to help them technically. They preferred to have such robot present with them rather than paper instruction manuals. As for the operation of such robots, the participants want a method of operation that is simple as a land-line phone or home appliances.

*4) Limitation of robot:* Throughout the PD workshops in Japan, although the participants discussed the design of the robots to solve their current social challenges, there were some limitations in these PD sessions. One was less concrete ideas about the robots to solve their challenges. For example, in the third round, the participants discussed how to improve sharing their experiences with people who are apart. In these discussions, they agreed that current online communication tools provide less information than in-person meetings, so they wanted new technology that can make them share more of their experiences with each other. However, while there were some remarks about specific experiences they would like to share, such as traveling or eating, they could not discuss or propose concrete and specific solutions to resolve their such challenges. P3_*Japan*_ made the following remarks, “I want an exciting robot that could engage same tasks or events together, or an exciting tool that make everyone enjoyable time together, but I cannot describe or present it because it is quite difficult to shape this idea concretely…”

In addition, some participants felt frustrated that their families and friends could not use the current social technologies to connect with them online, even as they succeeded in adopting such new technologies. P10_*Japan*_ said, “People around my age and older are very timid about new technology... I tried to explain to them that it is easy to use online tools like Zoom, but they just said to me ‘*no no no no'* and ‘*impossible!!'* without touching these tools…” Since the participants in these PD workshops actually have certain levels of literacy of such technologies, they were frustrated by the differences in literacy levels among their families and friends who are apart.

## 5. Discussion

To understand middle-aged and older adults' difficulty with current social technologies and develop ideas for designing socially facilitative robots to address their needs, we conducted PD workshops: three sessions in the US with seven middle-aged and older adult participants, and four session in Japan with twelve participants. Overall, participants were eager to discuss how robots might facilitate social interactions, while still harboring some reservations based on their negative experiences with current social technology and concerns about ease of use. Although we conducted the workshops during the COVID-19 pandemic, many findings generalized to other situations related to social isolation, such as older adults living alone. In this section, we present the major themes of our PD workshops while highlighting areas of focus for future PD and user-centered design intended to promote middle-aged and older adults' use and long-term acceptance of socially facilitative robot technologies.

### 5.1. Challenges with social technology

US and Japanese participants alike recognized some benefits of technology-mediated social interactions; however, they generally agreed that these mediums imperfectly replicated key aspects of in-person interactions. Both social and technical limitations of current social technology frustrate them, with all participants presenting ways in which technology-mediated interactions are inferior to traditional means of socializing. A lack of critical social cues, such as intonation and a sense of being together with others in a shared space, frustrated them over repeated interactions. Conversational cues that exist during in-person interactions, such as leaning in to indicate that you would to speak and timing of when to respond to a person, ultimately break down into cross-talk and/or stilted conversations. These social distractions detract from the quality of their interaction.

Technical issues such as dropped calls, audio lagging or breaking up, video freezing, and the lack of a turn-taking mechanisms in video calls compounded their frustration. These technical issues did not merely aggravate participants, but were a main cause for concern as our participants considered the difficulties of connecting with users, such as their parents, who may be even older or less technically proficient than them. It can be a burden to people of our participants' generations to have to teach their parents both how to technically use the social technology (what buttons to press when) but also address the communication etiquette of that social technology, particularly for the Japanese participants.

While participants related several social challenges they faced when keeping up with existing friends, our middle-aged and older adult participants emphasized the challenge of building new connections with others. These challenges were particularly salient during times of social distancing due to the COVID-19 pandemic, as traditional means of socializing familiar to older adults were limited, but would also extend to situations in which older adults are confined to their homes, hospitals, or elder care facilities.

### 5.2. Concepts for socially facilitative robots

Participants expressed general excitement for the potential of robots to facilitate higher quality social interactions between people. As shown in the conversational flow diagrams in Figures 1–3, 5–8 representing the PD sessions in the US and Japan respectively, our method was then successful at encouraging participants to develop and build upon a wide range of novel concepts: from robots dependent on physical embodiment in a shared space, to new applications of artificial intelligence, to representing social companionship through “body warmth.” In the US, the top three robotic concepts were telepresence robots, distancing robots, and AI to extend capability; in Japan, they were pet robots, sharing experiences, and easy operation to solve presence problems. Although these concepts may seems distinct, we find complimentary themes in places.

In the first theme, we note that *there are opportunities for robots to both socially connect people in the capacity of a tool* (i.e., telepresence, artificial intelligence enhanced social skills), *and also to use its own presence for a person to interact directly with* (i.e., pet robot, instructor robot). The telepresence robots conceived by participants in the US PD groups directly addressed their concerns with both maintaining current relationships and forming new connections while geographically removed from other people; positive discussions around these robots confirm findings of older adults' interest in the GiraffPlus telepresence robot in a PD workshop conducted in prior research (Van Baarsen et al., 2001). A review of mobile telepresence robot research prior to and during the global pandemic also indicates that the theme of telepresence would likely have scaled beyond the limited number of participants in our PD sessions (Isabet et al., 2021).

Participants in both countries noted that, not only is it difficult to meet new people when socially isolated, it can be difficult to trust who they are and have enough information to start a conversation. Artificial intelligence (AI) is already used in suggesting likely friends based on a person's social network, but can extend to be the entity to make that introduction and recommend topics of common interest. Participants' concepts of AI programs were targeted at improving the quality of existing social interactions. These applications might help address ongoing social issues such as avoiding potentially offensive language or offering reminders of scheduled times to meet with friends. This could help people in online situations regardless of age, and possibly provide different suggestions across different contexts to help people write more professional emails for work or more friendly emails for reaching out to new acquaintances.

Also, the idea of an “instructor robot” came from the PD sessions in Japan. Participants expressed a concern for making interactions with robots as easy as possible, particularly for their own older parents. If their parents are alone and need assistance operating a social technology, they envisioned that the instructor robot would teach and guide their parents on how to use it. This functionality speaks to lowering the technology savviness barrier and making it more accessible, which is another way to connect people socially.

If perceived as an agent, an instructor robot can also be the target of social companionship intended or otherwise. Japanese participants very much wanted pet robots to keep their parents from feeling lonely; all 4 Japanese PD groups discussed functionality they desired, so we believe that this concept would maintain its popularity if we had hosted additional PD sessions in Japan. Despite there being a plethora of academic research on robotic pets and commercially available ones (Eachus, 2001; Usui, 2011; Katsuno and White, 2022), none of the US PD groups discussed pet robots; however, in our previous work, middle-aged and older adults did show interest in pet robots in the one-on-one interviews (Ling et al., 2022). The Japanese participants had difficulty in expressing with the easy to use instructor robot and how to better share their experiences. However, they were very clear in articulating concrete capabilities a pet robot should have, including “body warmth” which can comfort their parents (Walsh, 2009; Zilcha-Mano et al., 2012).

In the second theme, participants in both the US and Japan noted that *the experience of sharing was lacking*. In the case of the US, participants wanted to “be there” meaning to travel, whereas the Japanese participants wanted a better way to share out their experiences with others. The US participants' interest in the possibility of combining robotics with emerging technologies like VR and AR present a novel finding from this work on an additional area of interest to this population. These technologies could address users' repeated observations that current two-dimensional video calls lack the spatial sense and full-body communication integral to meaningful social interactions.

A “distancing robot” could allow people to safely socialize in the same shared space. Participants in the US PD sessions thought a distancing robot could enable the development of new social connections between people even when physical contact was limited. For instance, during a global pandemic, group meetings were considered unsafe. Nonetheless, with a distancing robot, individuals could share the same physical space while maintain social distancing, by having the robots delivering items between people. With such a robot, club meetings of people with shared interests could continue in a physically shared space, rather than merely online, potentially enabling that group to facilitate new connections. Although the overall sample size of our PD sessions is small, it is likely that distancing robots would have continued to be a prevalent theme, as robotics researchers had also begun prototyping this concept (Sathyamoorthy et al., 2020, 2021; Somaldo et al., 2020).

While the middle-aged and older adults in this study were interested in the potential of socially facilitative robots, some skepticism remained. Similar to their discussion on perceived limitations of current social technology, participants also imagined limitations of the robots they brainstormed during this session. Their concerns emphasize the need for robot designers to involve middle-aged and older adults at every stage of the design and development process, to ensure that their needs are being met and that this population are willing to use new technology.

While not all brainstormed concepts may be immediately feasible, these PD workshops provide insights for designers based on insights into middle-aged and older adults' expectations and reservations around the capabilities of social robots. Our participants emphasized the importance of perceived ease-of-use and reliability when making decisions in new technology use. This poses a unique problem for social technology, as users must consider not only their own technical proficiency, but the capabilities of those they plan to connect with. Older adults may prefer natural language voice interfaces over other input modalities, as this leverages their understanding of conversations. Finally, to avoid undue anxiety, designers must clearly communicate that the purpose of socially-facilitative robots. In the US, the goal is not to replace, but rather aid, human social interactions, while in Japan, the goal is to for robots to be a stand-in companion in the absence of people.

### 5.3. Limitations

The online nature of this study presented a notable limitation to the current research. The participant sample all had access to the Internet and exhibited some degree of technical competency, as they were able to participate in a group online video call. This prevented us from studying populations of older adults who lack either perceived technical competency or access to the internet altogether. A future in-person workshop may better accommodate this population and produce new insights.

Participants did not discuss or build upon each others' ideas as much as we expected. We found that US participants spoke more than the Japanese participants, which could be attributed to a number of factors including participants' lack of confidence, insufficient rapport among PD groups, or unintended change in facilitation style when culturally adapted for use in Japan. Another factor impacting participation in discussion might the be platform of choice used to host these online participatory design sessions and people's familiarity with using the platform and associated social etiquette (e.g., mute when not speaking, unmute to speak, raise your hand). We used Zoom. All US participants used Zoom (*n* = 5) or equivalent video calling apps (*n* = 2), which are typically used on a computer, laptop, or larger tablet. Only 4 of the 12 Japanese participants called out Zoom, and majority (*n* = 8) used LINE on their smartphones. LINE is a mobile app that has an integrated video call feature and handles other forms of social content/connection, whereas Zoom's primary functionality is video calls on computers or tablet devices with groups of people, such as with our PD sessions. In addition, participants' educational backgrounds and health conditions, and the economic situation, may have affected their opinions in PD sessions so future studies should examine other populations.

In the future, we recommend that online PD sessions should have minimally 3 or ideally 4 participants; we believe this will reduce the perceived burden of participants feeling pressure to perform, like too much is riding on how their responses. We posit that a having 2 participants, as we did for US PD sessions 2 and 3, makes them feel culpable for 50% of the content, whereas with 4 people, their individual responses are only 25%. 3 or 4 participants helps build a wider variety of ideas, while still maintaining enough time for sharing out without having to significantly extend the duration the PD session.

Further, the current workshop consisted of a relatively open brainstorm session of imagined robot functions and features which is useful at the beginning of a project. Researchers should conduct future PD workshops at different stages of the design process, such as when drafting more specific robot designs or testing physical prototypes. There may be ethical concerns about socially facilitative robots to reduce social isolation for older adults in the US and Japan (e.g., privacy). We have mentioned these concerns as participants brought them up throughout the sessions, but researchers should conduct future work to examine what concerns may arise as the technologies are implemented.

Finally, our findings should be considered with respect to people's evolving perceptions, attitudes, and behaviors over time during the COVID-19 pandemic. Kim and Crimmins (2020) conducted a study over the first 3 months of the pandemic in the US and in which preventative and protective behaviors people engaged, including wearing a mask, washing hands, avoiding crowds and eating at restaurants, postponing or canceling social gatherings, and avoiding hosting guests in their homes. They found that as people learned over time about the risks, they adopted more of these preventative and protective behaviors. The 3 US participatory design groups were held in November and December 2020, approximately 6 months after the start of the pandemic. At that time, people were cautioned against holiday season travel and large gatherings (Abidi and Gramlich, 2020). The 4 participatory design groups in Japan were held in August and September 2021. Kim and Crimmins noted the likely possibility that people could not engage in these protective and preventative behaviors for the long term. Globally, people had been dealing with COVID-19 for well over a year and acclimated to the “new normal” (Jamaludin et al., 2020).

## 6. Conclusion and future directions

While this study occurred during a time of pronounced social isolation due to the COVID-19 pandemic, many of the challenges discussed relate to social issues participants experienced prior to the pandemic, and which will be ongoing issues for older adults in the future. Many issues were not caused, but rather exacerbated, by novel social distancing measures. For example, P7_*US*_ expressed trust issues with the concept of online dating prior to the pandemic, but these issues became more salient as the pandemic restricted other potential options for meeting new romantic partners. Further, many of the challenges discussed here will remain pertinent after social distancing requirements are lifted, as older adults may live far apart from their loved ones and social interactions continue to move online.

Recent research has emphasized the importance of directly involving end users of technology as collaborators in the design process (Chen et al., 2018). It is crucial that older adults are involved in the design of robots intended to meet their particular needs (Chopik, 2016). Our research adds to the literature by (1) tailoring a PD approach to create a novel grounded approach for people to reflect on their own life experiences prior to giving them an abstract topic to discuss, such as robots, and (2) examining the social needs of middle-aged and older adults and how socially facilitative robots could be applied to address their self-identified needs. Our findings underscored the importance of adopting a user-centered approach for socially facilitative robot design for older adults.

## Data availability statement

The raw data supporting the conclusions of this article will be made available by the authors, without undue reservation.

## Ethics statement

The studies involving human participants were reviewed and approved by Institutional Review Board of New Mexico State University; Ethics Committee on Human Research of Meiji University. The patients/participants provided their written informed consent to participate in this study.

## Author contributions

HP and RA were the primary authors for the participatory design groups run in the United States. TK is the primary author for those run in Japan. SS created the conversational flow diagrams. All authors contributed to the analysis and/or writing of this paper. All authors contributed to the article and approved the submitted version.

## Funding

This work was supported and funded by the Toyota Research Institute.

## Conflict of interest

Author KT receives a nominal financial award for publishing academic papers. The remaining authors declare that the research was conducted in the absence of any commercial or financial relationships that could be construed as a potential conflict of interest.

## Publisher's note

All claims expressed in this article are solely those of the authors and do not necessarily represent those of their affiliated organizations, or those of the publisher, the editors and the reviewers. Any product that may be evaluated in this article, or claim that may be made by its manufacturer, is not guaranteed or endorsed by the publisher.

## Footnotes

^1^The New Mexico State University Institutional Review Board approved the US PD workshop on August 27, 2020; “Technology and Social Activities III (Focus Group), ID: 20312.” The Ethics Committee on Human Research of Meiji University approved the Japanese PD workshops (Application Number: 2019-39).

^2^ https://line.me/en/
